# All Is Relative—A Call for Considering “Physiologically Informed” Control Conditions to Improve the Mechanistic Understanding of the Effects of Physical Exercise on Cognition

**DOI:** 10.1111/sjop.70101

**Published:** 2026-04-26

**Authors:** Fabian Herold, Benjamin Tari, Sylwester Kujach, Olivier Dupuy, C. Shawn Green, Thomas Gronwald, Matthew Heath

**Affiliations:** ^1^ HMU Health and Medical University Erfurt Erfurt Thuringia Germany; ^2^ Institute of Sport, Exercise and Health University College London London UK; ^3^ Department of Physiology Gdansk University of Physical Education and Sport Gdansk Poland; ^4^ Department of Physiology, Faculty of Medicine Medical University of Gdansk Gdansk Poland; ^5^ Laboratory MOVE, Faculty of Sport Sciences University of Poitiers Poitiers France; ^6^ Department of Psychology University of Wisconsin Madison Wisconsin USA; ^7^ Institute of Interdisciplinary Exercise Science and Sports Medicine MSH Medical School Hamburg Hamburg Germany; ^8^ G‐Lab, Faculty of Applied Sport Sciences and Personality BSP Business and Law School Berlin Germany; ^9^ School of Kinesiology, Faculty of Health Sciences University of Western Ontario London Ontario Canada; ^10^ Canadian Centre for Activity and Aging University of Western Ontario London Ontario Canada; ^11^ Graduate Program in Neuroscience University of Western Ontario London Ontario Canada

**Keywords:** neuroscience, physical activity, study design, training, triangulation

## Abstract

There is a growing interest in elucidating the mechanisms that drive the benefits of physical exercise on cognitive performance. A key element for a better understanding of a particular phenomenon (e.g., the mediators of the exercise‐cognition interaction) is the selection of an appropriate control condition/group as the basis for causal inference. In contemporary practice, control conditions/groups used in exercise‐cognition research can be broadly categorized as related to (i) study design, (ii) level of energy expenditure, and (iii) level of cognitive engagement. Although such control conditions are valuable for reducing the effects of specific sources of bias (e.g., time or placebo effects), their potential to advance our mechanistic understanding is limited. To address this research gap, the present article proposes and discusses the application of “physiologically informed” control conditions by narratively summarizing the current evidence concerning “physiologically informed” control conditions in acute exercise‐cognition studies, wherein specific physical exercise‐induced physiological responses (e.g., increases in cerebral blood flow or peripheral blood lactate concentration) are mimicked by nonexercise experimental manipulations (e.g., through inhaling hypercapnic gas mixture or infusion of lactate at rest). Based on our narrative evidence synthesis, we discuss how “physiologically informed” control conditions can serve as a valuable approach to strengthen causal interferences by allowing for a better isolation of cognitive benefits that can be “solely” attributed to specific physical exercise‐induced physiological changes. As applying “physiologically informed” control conditions can advance knowledge generation on the physiological mechanisms that drive the positive effects of physical exercise on cognition, we advocate for a more widespread use of these control conditions in future research practice.

## Summary

- Appropriate control conditions are crucial for establishing causal relationships in exercise‐cognition studies.
- “Physiologically informed” control conditions, wherein physical exercise‐induced physiological responses are mimicked through nonexercise experimental manipulations, have been largely overlooked in exercise‐cognition research.
- Implementing “physiologically informed” control conditions in future exercise‐cognition studies will enable a better isolation of cognitive benefits that can be “solely” attributed to specific physiological changes caused by physical exercise.
- A more widespread use of “physiologically informed” control conditions in future research practice will deepen our mechanistic understanding of the exercise‐cognition link.

## 1 Introduction

**FIGURE 1:**
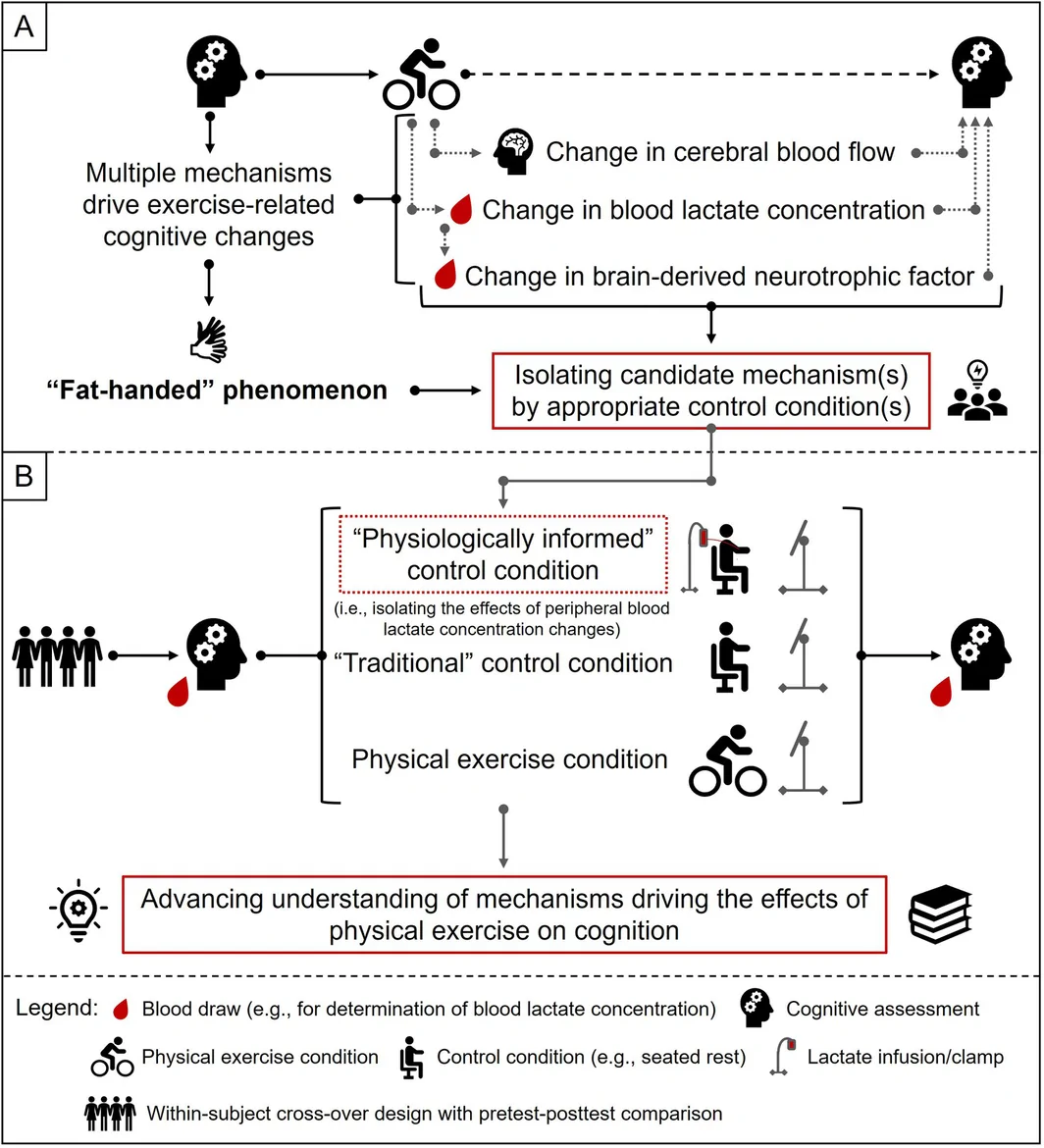
(Panel A) Schematic illustration of selected physiological candidate mechanisms that might drive the positive effects of physical exercise on cognitive performance. We also present how isolating physiological candidate mechanisms can be used to account for the “fat‐handed” phenomenon. (Panel B) An exemplar study design (i.e., within‐subject cross‐over design with pretest–posttest comparison, as recommended in Herold et al. 2021; Pontifex et al. 2019), in which a “physiologically informed” control condition (i.e., lactate infusion/clamp; matched to lactate changes in physical exercise condition) is used in addition to a “traditional” control condition (i.e., contact‐control condition—sitting while watching a neutral video) and a physical exercise condition (e.g., inducing comparable peripheral lactate concentration changes as in “physiologically informed” control condition) to more rigorously isolate the effects of peripheral blood lactate concentrations on changes to cognitive performance. Please note that watching the neutral video during all conditions is used to control for the level of cognitive engagement. Furthermore, the participant should be seated on the same device (e.g., cycle ergometer) during all conditions, with the latter being displayed differently in the Figure to avoid a misleading interpretation (e.g., that the participant is asked to exercise in the control conditions). In addition, advancing the study design by additional conditions in which, for example, participants are provided with a sham infusion or conduct a sham physical exercise protocol is another option to reduce the effects of potential confounders (e.g., placebo effects).

There is growing evidence that acute (Chang et al. 2025; Garrett et al. 2024) and chronic (Erickson et al. 2019; Ludyga et al. 2020) physical exercise—as planned and structured forms of physical activity (Bishop et al. 2025; Caspersen et al. 1985)—improve cognitive performance. However, the mechanism(s) that drive this positive effect are not fully understood (Herold, Müller, et al. 2019; Herold, Törpel, et al. 2019; Pontifex et al. 2019; Stillman et al. 2016; Stillman et al. 2020). It is assumed that such positive effects are mediated by changes on multiple levels (see Figure 1A) such as (i) molecular and cellular level (e.g., biochemical changes such as increased blood lactate concentration), (ii) functional and structural brain level (e.g., change in cerebral blood flow or gray matter volume in specific brain regions), and (iii) socioemotional and/or psychophysiological level (e.g., changes in sleep and/or arousal) (for reviews see Herold, Törpel, et al. 2019; Stillman et al. 2016, 2020). To elucidate how multilevel alterations can explain the positive effects of physical exercise on cognition, several approaches, including but not limited to statistical (e.g., application of mediation analysis) or study design‐related ones (e.g., selection of an appropriate control condition/group), can be used.

Given that our understanding of the effects of physical exercise on cognition is typically informed by statistical comparisons of an exercise intervention/group to a control condition/group, selecting an appropriate control condition/group is critical for robust causal inference. As has been discussed for other application scenarios (i.e., cognitive training; for reviews see Green et al. 2014, 2019), a control condition/group is important to account for a set of different sources of confounders, including time, habituation/learning (Herold et al. 2021), and placebo effects (Beedie et al. 2018; Lindheimer, Szabo, Raglin, and Beedie 2020; Lindheimer, Szabo, Raglin, Beedie, Carmichael, and O'Connor 2020). Although selecting appropriate control conditions/groups is relevant for acute physical (i.e., a single bout of physical exercise) and chronic physical exercise studies (i.e., repeated bouts of physical exercise; Budde et al. 2016), this article primarily focuses on acute physical exercise studies because the use of “physiologically informed” control conditions is, to our knowledge, largely absent from chronic physical exercise studies. That being said, the assumptions presented in this article are, at least in part, also relevant for chronic physical exercise studies.

Contemporary research practice in the exercise‐cognition field—particularly studies investigating the effects of a single bout of exercise on cognition—has employed an approach to select the control condition/group based on a broad categorization that relates to (i) study design, (ii) level of energy expenditure, and (iii) level of cognitive engagement

- Concerning study design, using a within‐subject study with a randomized cross‐over design and a pretest‐posttest comparison is considered the most rigorous approach in this research field,^1^ depending on the research aim and context. This is because participants serve as their own controls, and thereby reduce the effects of potential participant‐specific factors (e.g., genotype) on outcomes. Furthermore, this type of design accounts for possible day‐to‐day variations in the reference measure of cognitive performance (e.g., baseline shift during pretest before each condition) that can arise from fluctuations in a variety of influencing factors, including learning/habituation effects, and/or changes in other participant‐specific factors (e.g., motivation, mood or hydration status) (for reviews see Herold et al. 2021; Pontifex et al. 2019). It is, however, worth noting that such an approach comes at the cost of being more resource‐consuming, more burdensome for the participant (e.g., multiple lab visits/sessions), and the risk of more pronounced learning/ceiling effects due to the repeated administration of the cognitive tests (Herold et al. 2021; Pontifex et al. 2019). Another study design‐related approach is the *baseline control*, wherein cognitive baseline performance is either assessed on a separate day or on the same day preceding the experimental conditions. However, the data from the baseline control is not used as a pretest during statistical analysis (Pontifex et al. 2019); rather, it provides information to account for participant‐specific differences in baseline cognitive performance in statistical analysis. The latter is an important moderator of the effects of acute (Drollette et al. 2014; Ishihara et al. 2021; Mou et al. 2023; Yamazaki et al. 2018) and chronic physical exercise (Ishihara et al. 2020) on cognitive performance. For example, baseline control can be used to assess whether participants with lower baseline cognitive performance show a greater cognitive performance improvement following acute or chronic physical exercise than those with a higher baseline performance.
- Concerning energy expenditure, frequently employed options include (a) an *active control condition/group*, involving physical activity (i.e., defined as any skeletal muscle‐induced bodily movement (Caspersen et al. 1985) that results in energy expenditure ≥ 1.5 metabolic equivalent of task (MET; Falck et al. 2023)); this might include but is not limited to a *sham control condition/group* which can be characterized by physical exercise with an insufficient loading to trigger a meaningful response (e.g., stretching or toning exercise), or (b) a *nonexercise control condition/group* which is often also referred to as a passive controlled condition/group and is synonymous with using sedentary behaviors (i.e., defined as waking behavior characterized by a low energy expenditure ≤ 1.5 MET while in a sitting, reclining, or lying posture; Tremblay et al. 2017) like seated rest as a control (Herold et al. 2021; Pontifex et al. 2019).
- Concerning the level of cognitive engagement, possibilities include (i) a *contact‐control* condition/group (i.e., also is referred to as [*cognitively*] *active* or *engagement control*) such as watching television, reading, or a sham control, or (ii) a *non‐contact control* condition/group (i.e., also referred to as [*cognitively*] *passive* or *disengagement control*) like seated rest on a cycle ergometer without an explicit cognitive task (Green et al. 2014, 2019; Herold et al. 2021; Pontifex et al. 2019).

It is important to clarify that the meaning of the attributes “active” and “passive” used to characterize the control conditions/groups can vary, depending on whether they are used to refer to the energy expenditure or the level of cognitive engagement inherent to the control activity (Green et al. 2014; Herold et al. 2021). To avoid confusion, those classifications should not be conflated and used with appropriate care in communication, especially as they are complementary rather than mutually exclusive. For example, being in a seated position on a cycle ergometer while concurrently reading a book can be considered “physically” passive while “cognitively” active. In contrast, using cycling as a form of sham control (i.e., cycling at lower intensity than in the intervention condition/group) while having no contact with others (e.g., the experimenter) can be seen as “physically” active while being “cognitively” passive. Although there is no “one‐size‐fits‐all” control condition in exercise‐cognition studies, as the latter depend on the study's context and aims (Green et al. 2014, 2019; Herold et al. 2021; Pontifex et al. 2019), performing the “control activity” in both the control and intervention condition/group (e.g., watching a neutral video during seated rest and while cycling; as done in Ellemberg and St‐Louis‐Deschênes 2010; Ludyga et al. 2023) might be a valuable and straightforward approach to isolate physical exercise‐related effects (Herold et al. 2021; Pontifex et al. 2019).

Comparable to acute physical exercise, there is also a discourse regarding the appropriate control condition/group in studies investigating, for example, the chronic effects of physical exercise on cognition. This might include, but is not limited to, the opinion that physically active and fit individuals should be used as controls (Booth and Lees 2006; Buford and Manini 2010) or the ethical aspects related to using a physically inactive control groups (Gambassi and Schwingel 2026). Resting on the assumption that causality can be established by increasing or decreasing the exposure to a stimulus, it has been proposed that a purposeful and time‐constrained reduction of the regular level of physical activity (e.g., daily step count reduction and detraining), as a complementary approach to the experimental increase in regular physical activity, may serve to provide a more comprehensive understanding of the influence of physical exercise, including its mechanisms, on cognition (Herold, Ludyga, et al. 2025). However, empirical studies utilizing this approach as an intervention or a control condition, especially with regard to cognition and brain health, are relatively scant (Herold, Ludyga, et al. 2025; Pinto et al. 2023).

Taken together, when one aims to make robust causal inferences, selecting an appropriate control condition/group is crucial to isolate the specific effects (e.g., by controlling for the level of cognitive engagement) by, ideally, accounting for all possible confounders of interest. Here, “of interest” is key, as the goal of a given study strongly determines what potential confounds may be “of interest.” For example, work in the broader cognitive intervention field (Green et al. 2014, 2019) has differentiated between “efficacy” or “effectiveness” studies and “mechanistic” studies. In efficacy/effectiveness studies, the goal of an intervention is to demonstrate that an intervention produces benefits above and beyond some minimum types of confounds, such as expectations or placebo effects (Ziv et al. 2024), although the placebo effect is unlikely to fully explain the positive effects of acute and chronic physical exercise on cognition (Oberste et al. 2017; Stothart et al. 2014). However, such efficacy/effectiveness studies do not (and cannot) seek to understand “why” the intervention produces an effect (i.e., they simply aim to establish whether an effect is present or not). Indeed, addressing “why” an effect occurs is the primary interest of mechanistic studies, which are the key concern of this article. Moreover, making causal inferences regarding human behavior, including driving mechanism(s), is challenging (Bailey et al. 2024). Part of this challenge can be drawn from the fact that an (exercise) intervention often does not only lead to a change in a single variable, as typically intended, but simultaneously leads to nonintended changes in other variables that may be causally related to a specific outcome (Bailey et al. 2024; Eronen 2020). This phenomenon also applies to the field of exercise‐cognition, especially to the mechanism(s) mediating these effects. This is because physical exercise typically triggers a myriad of psychophysiological responses that may, to some degree, be related to a particular outcome (i.e., cognition; see Figure 1A and for reviews Stillman et al. 2016, 2020).

Considering control conditions/groups that allow for better isolation of the effects of specific candidate mechanisms has the potential to advance our mechanistic understanding of the exercise‐cognition interaction. In this context, we propose that future mechanistic studies should consider “physiologically informed” control conditions. These can be operationally defined as control conditions in which a given physiological response typically triggered by acute physical exercise and associated with cognitive performance changes can be induced without engaging in physical exercise (i.e., through nonexercise experimental manipulations). For the remainder of the article, we intentionally use the term “physiologically informed” in a broader sense to ensure inclusiveness (e.g., to include physiological processes related to motor control and movement execution). As we will show below, such “physiologically informed” control condition(s) might yield complementary evidence compared to those “traditionally” used and may serve to advance our understanding of the causal mechanisms driving the effects of physical exercise on cognition.

## 2 “Physiologically Informed” Control Conditions/Groups

It is beyond the scope of this article to discuss all potential approaches for “physiologically informed” control conditions. Hence, in the next section, we narratively summarize and discuss key findings of selected studies. This section will highlight the potential of (i) hypercapnia, (ii) lactate infusion and clamp, (iii) passive exercise, (iv) motor imagery, and (v) electrical muscle stimulation as more appropriate contrasts than traditional control conditions for advancing our understanding of the physiological mechanisms driving the effects of acute physical exercise on cognition (for an overview see Table 1).

**TABLE 1:** Overview of selected types of “physiologically informed” control conditions, comparing them based on the following key dimensions: (i) target mechanism, (ii) invasiveness, (iii) feasibility, (iv) limitations, and (v) recommended use‐case. Please note that the ratings concerning these dimensions reflect general trends and that each of the “physiologically informed” control condition modalities has unique advantages and limitations for isolating the effects of the mechanistic pathway under investigation.

Modality	Target mechanism	Invasiveness	Feasibility	Limitations	Recommended use‐case
Hypercapnia	CBF elevation (via CO_2_‐driven vasodilation)	Noninvasive	Moderate	Affects arousal; safety considerations	Isolating CBF‐related effects
Lactate infusion/clamp	Peripheral blood lactate elevation	Invasive^a^	Low to moderate	Risk of adverse events; requiring supervision by a medical professional	Isolating lactate‐related effects
Passive exercise	Movement‐related sensory feedback	Noninvasive	High	Limited to simple movements (e.g., cycling)	Isolating movement‐related effects
Motor imagery	Motor‐control network activation	Noninvasive	Very high	Depends on participants' imagery ability	Isolating motor‐control‐related effects
Electrical muscle stimulation	Metabolic and sensory muscle activation	Noninvasive	Moderate to high	Physiological differences between involuntary muscle activation patterns compared to voluntary ones; discomfort is possible	Isolating voluntary muscle activation‐related effects

Abbreviations: CBF, cerebral blood flow; CO_2_, carbon dioxide.

^a^ Indicates that typically invasive approaches are used (e.g., via intravenous infusion; Coco et al. 2009; Kemna et al. 2025; Schiffer et al. 2011), while noninvasive alternatives are available (e.g., via a drinking beverage entailing a lactic acid solution; Cho et al. 2020).

### 2.1 Hypercapnia

Acute physical exercise results in an increase in cerebral blood flow (CBF; e.g., as estimated via transcranial Doppler ultrasound), which follows a U‐shaped timecourse during acute physical exercise (Ogoh and Ainslie 2009; Smith and Ainslie 2017) and typically returns to baseline levels ~30 min after exercise cessation (Kennedy et al. 2022; Mulser and Moreau 2023). Such acute physical exercise‐related changes in CBF are hypothesized to mediate cognitive performance improvements (Pontifex et al. 2019), although exercise‐related changes in global CBF alone may not fully explain improvements in cognitive performance due to the complex nature of neurobiobehavioral associations (Hashimoto and Ogoh 2025). However, in the context of an exercise‐mediated increase in CBF improving executive function (EF), the inclusion of a hypercapnic condition represents an important control because breathing a gas mixture with a higher‐than‐atmospheric concentration of carbon dioxide (CO_2_: i.e., a hypercapnic environment) triggers rapid chemoreceptor‐induced vasodilation that increases CBF (Ainslie and Duffin 2009; Danek 2026; Hoiland et al. 2019). Hypercapnia‐related changes to CBF are diffuse across the human brain (Ito et al. 2003) and include brain regions associated with acute physical exercise‐related changes in cognitive performance (e.g., frontal cortex; for reviews see Basso and Suzuki 2017; Herold, Aye, et al. 2020; Herold et al. 2018). However, studies using hypercapnia as a “physiologically informed” control condition to isolate the effects of a change in CBF on acute physical exercise‐induced cognitive improvements are rare, with notable exceptions (Shoemaker et al. 2020; Tari et al. 2020). For example, a within‐subject study with pretest‐posttest comparisons showed that a 10‐min hypercapnic control condition (i.e., sitting on a cycle ergometer while breathing a gas mixture of 5% CO_2_, 21% O_2_, 74% N_2_) induced a postexercise EF improvement (i.e., assessed via the antisaccade task) comparable in magnitude to a time‐matched acute aerobic exercise condition (i.e., cycling) entailing a matched increase in CBF (Tari et al. 2020). Another within‐subject study entailed a seated resting control condition as well as an exercise condition (i.e., treadmill walking at submaximal and severe intensities). Here, condition‐specific end‐tidal CO_2_ concentrations were matched between and clamped across exercise conditions and during hypercapnia. The authors reported postintervention improvements in mental switching performance (i.e., assessed 2 min after intervention conditions with the pro‐ and antipointing task) compared to the pretest, regardless of the experimental condition (Shoemaker et al. 2020). Notably, however, this study did not observe a statistically significant correlation between intervention‐related changes in CBF and cognitive performance in either the control or exercise conditions (Shoemaker et al. 2020).

The findings of both studies point to postintervention benefits in EF (Shoemaker et al. 2020; Tari et al. 2020), and one study supports the idea that acute changes in CBF are associated with an improvement in cognitive performance (Tari et al. 2020), whereas the other did not, at least based on the results of the correlation analysis (Shoemaker et al. 2020). Such divergent findings are likely related to differences in study design, particularly characteristics of the experimental manipulations (i.e., a 10‐min condition in a laboratory session (Tari et al. 2020) versus several experimental manipulations in a laboratory session no longer than 120 min (Shoemaker et al. 2020)). In any case, these results provide complementary and at least partly confirming evidence for the acute physical‐exercise literature reporting a positive correlation between an exercise‐induced increase in CBF and a concomitant benefit to cognitive performance (Shirzad et al. 2022; Tari et al. 2020). Based on these observations, inducing hypercapnia (e.g., during seated rest) can be considered a promising noninvasive approach to “mimic” an acute physical exercise‐induced increase in CBF without actually engaging in acute physical exercise. This is true at least within a specific exercise intensity range because a decline in CBF has been observed at higher exercise intensities (Ogoh and Ainslie 2009; Smith and Ainslie 2017). Mimicking the latter may require inducing a state of hypocapnia, wherein cerebral vasoconstriction elicits a CBF reduction (Ainslie and Duffin 2009). However, hypocapnia also leads to better cerebral oxygen extraction (Jiang and Lu 2022), which may offset, at least to some extent, the potentially negative effects of an acute hypocapnia‐induced reduction of CBF on cognition. Such a complex relationship between CBF, other physiological processes, and cognition should be considered when using hypocapnia as an experimental manipulation to induce CBF changes (i.e., as a “physiologically informed” control condition).

Taken together, mimicking acute physical exercise‐induced changes in CBF (e.g., by inhaling hypercapnic gas at rest) allows for better isolation of CBF as a physiological candidate mechanism for cognitive improvements. The use of these control sessions can thus advance our mechanistic understanding of the exercise‐cognition interaction. To avoid adverse health‐related events when using this experimental manipulation as a “physiologically informed” control condition, safety recommendations regarding the application of hyper‐ and hypocapnia should be considered (e.g., for reviews see Ainslie and Duffin 2009; Danek 2026; Gitelman et al. 1991).

### 2.2 Lactate Infusion and Clamp

Peripheral blood lactate shows an exercise intensity‐related increase and is a biomolecule known to cross the blood–brain barrier via monocarboxylate transporters (Halestrap and Wilson 2012; Pierre and Pellerin 2005; Quistorff et al. 2008; Riske et al. 2017), with peripheral plasma lactate levels and those observed in specific brain regions (i.e., dorsomedial prefrontal cortex/dorsal anterior cingulate cortex) showing a positive association (Clairis et al. 2024). Moreover, lactate serves as an important fuel for cognitive processes, particularly during periods with heightened bioavailability (e.g., during or shortly after exercise; for reviews see Brooks 2018, 2020; Brooks et al. 2021, 2023; Hashimoto et al. 2021; Quistorff et al. 2008; Riske et al. 2017; Taher et al. 2016). Several within‐subject studies with a randomized cross‐over design and pretest–posttest comparisons investigated whether acute physical exercise‐induced changes in peripheral blood lactate concentration were associated with postexercise cognitive improvements. The studies assessed responses to acute physical exercise protocols with varying durations and intensities: (i) 14 (Nunes Pereira Oliva et al. 2023) or 20 min (Ballester‐Ferrer et al. 2022) of moderate‐intensity continuous cardiorespiratory exercise, (ii) 14 (Nunes Pereira Oliva et al. 2023), 20 (Ballester‐Ferrer et al. 2022), or 33 min (Tsukamoto et al. 2016) of high‐intensity interval exercise; or (iii) 10 min of sprint‐interval training (Herold et al. 2022). In general, those studies observed a positive association between acute physical exercise‐related changes in blood lactate concentration and attention (Herold et al. 2022) and EF performance (Ballester‐Ferrer et al. 2022; Nunes Pereira Oliva et al. 2023; Tsukamoto et al. 2016). These results were found with delays between exercise cessation and cognitive testing of 10 (Herold et al. 2022), 15 (Ballester‐Ferrer et al. 2022), and up to 50 min (Tsukamoto et al. 2016, but see Nunes Pereira Oliva et al. 2023). Notably, a recent systematic review summarizing the current state of evidence concluded that peripheral blood lactate concentrations measured immediately after an acute bout of high‐intensity interval exercise explained 14%–17% of variance in inhibitory control performance assessed 30 min after exercise cessation (Jacob et al. 2023). Moreover, a study in healthy young adults using a between‐subject pretest–posttest design found that acute physical exercise‐induced increases in peripheral blood lactate following a 25‐min bout of moderate‐intensity continuous cardiorespiratory exercise—performed either alone or combined with resistance exercise—mediated improvements in cognitive flexibility when assessed immediately after exercise cessation (Li et al. 2024). In interpreting these results, it is important to recognize that the role of peripheral lactate in producing a postexercise cognitive benefit is equivocal. For example, another within‐subject study with a randomized cross‐over design with pretest–posttest comparison did not observe a mediation effect of peripheral lactate among healthy young adults. In that study, inhibitory control was measured immediately before and ~11 min after a 20‐min bout of exercise involving 5 min of sprint interval training (Yamada et al. 2023). Furthermore, a between‐subject study with pretest–posttest comparison in healthy young adults did not observe a positive association between acute physical exercise‐induced changes in peripheral blood lactate concentration and EF and memory performance in response to a 35‐min bout of light‐, moderate‐, or high‐intensity cardiorespiratory exercise (Oberste et al. 2016). Consistent with this observation, a within‐subject study with pretest–posttest comparison, which investigated the effects of an acute bout of exhaustive physical exercise in healthy young adults, did not demonstrate a statistically significant association between acute physical exercise‐related changes in peripheral blood lactate concentration and behavioral performance in an auditory oddball task (Abhishek et al. 2025). However, based on a correlation analysis, this study reported a positive association between acute physical exercise‐related changes in peripheral blood lactate concentration and specific neuroelectric indices (i.e., N2P3 peak‐to‐peak‐amplitude recorded during the oddball task), but the same data yielded only a trend towards the conventional threshold of significance (i.e., typically defined by *p* ≤ 0.05) when analyzed with a linear regression (Abhishek et al. 2025). Notably, another within‐subject study with a pretest–posttest comparison observed in healthy young adults a statistically significant correlation between acute physical exercise‐related changes in peripheral blood lactate levels and specific neuroelectric indices recorded during a visual‐evoked potential paradigm (Abhishek et al. 2024). Specifically, a decrease in P100 latency immediately after an acute bout of exhaustive physical exercise and an increase in N145 latency at the 10‐min follow‐up assessment were shown (Abhishek et al. 2024). However, on a behavioral level, other studies involving healthy young (Coco et al. 2009) and older adults (Coco et al. 2020) have reported a negative association between changes in peripheral blood lactate concentration and attention (Coco et al. 2009) and EF performance (Coco et al. 2020) when the latter were assessed briefly after (i.e., ≤ 5 min) an exhaustive physical exercise bout (i.e., a maximal multistage discontinuous incremental cycling test); such negative effects dissipated at later follow‐ups, conducted 10 (Coco et al. 2009) or 15 min (Coco et al. 2020) after exercise cessation.

Taken together, there is equivocal evidence as to whether peripheral blood lactate concentration can be considered as a mediator of acute physical exercise‐related cognitive improvements. It is likely that the inconclusive nature of the evidence is at least partly related to the sizeable heterogeneity in key study characteristics such as exercise intensity and postexercise assessment time points, moderators known to impact the expression of postexercise cognitive benefits (for review see Chang et al. 2012). Regardless, there is sufficient evidence to suggest that the impact of serum changes in lactate concentration deserves further attention as a potential mechanism supporting a postexercise cognitive benefit.

The current evidence on the association between acute physical exercise‐related changes in peripheral blood lactate concentration and cognitive performance is primarily informed by correlational analyses (Abhishek et al. 2025; Ballester‐Ferrer et al. 2022; Coco et al. 2020; Herold et al. 2022; Nunes Pereira Oliva et al. 2023; Oberste et al. 2016; Tsukamoto et al. 2016). When considering complementary approaches that allow for more robust causal inference, isolating the effects of peripheral blood lactate on cognition (e.g., by a “physiologically informed” control condition) may be a promising method for advancing our mechanistic understanding of the exercise‐cognition interaction. A potentially useful technique to isolate the effects of blood lactate to mimic its acute physical exercise‐related increase at rest includes in vivo infusion of lactate (e.g., via a sodium lactate infusate with or without a tracer; Emhoff and Messonnier 2023), or its extension, the so‐called “lactate clamp.” The latter permits the maintenance of a precise steady‐state blood lactate concentration even during prolonged intervention periods. The maintenance of these concentrations is based on frequent measures via a portable analyzer that guides the adjustment of the lactate infusion rate (Emhoff and Messonnier 2023; Miller, Fattor, Jacobs, Horning, Suh, et al. 2002). For example, sodium lactate infusions have been used to investigate the effects of blood lactate changes on cognitive performance (i) during rest in patients with brain injury undergoing neurosurgical treatment (Bisri et al. 2016) or older adults with Alzheimer's Disease (Kálmán et al. 2005), or (ii) during rest and physical exercise to study its role in metabolic (Boyd et al. 1974; Messonnier et al. 2013; Ryan et al. 1979) or respiratory responses (Ryan et al. 1979). The lactate clamp, which was pioneered in humans by Miller and colleagues (Miller, Fattor, Jacobs, Horning, Suh, et al. 2002), has been primarily used during rest and physical exercise conditions to understand the effects of lactate on cardiorespiratory (Miller, Fattor, Jacobs, Horning, Suh, et al. 2002), metabolic (Miller, Fattor, Jacobs, Horning, Navazio, et al. 2002; Miller, Fattor, Jacobs, Horning, Suh, et al. 2002), hematological (Miller et al. 2005), or catecholamine responses (Fattor et al. 2005).

Although the above‐discussed studies suggest that lactate infusion and lactate clamp protocols can be considered feasible techniques to better understand mechanisms in the broader field of (exercise) physiology, these approaches have been rarely used in studies in the exercise cognition field (e.g., as a “physiologically informed” control condition), with three notable exceptions (Coco et al. 2009; Kemna et al. 2025; Schiffer et al. 2011). First, a study involving six healthy young adults used an intravenous lactate solution infusion (i.e., 2 milliequivalents/ml lactate solution at a rate of 3 mg per kilogram bodyweight [kg/BW] in 1 min) while participants sat in a comfortable position in an armchair (Coco et al. 2009) in order to mimic acute physical exercise‐induced peripheral blood lactate changes. The study also aimed to investigate causality related to a decrease in attentional performance. Results showed that in response to the lactate infusion at rest, a decrease in attentional performance occurred (Coco et al. 2009). This observation does support the idea of a causal relationship between changes in peripheral blood lactate concentration and worse cognitive performance (i.e., attentional performance), and complements evidence from previous correlational studies (for review see Jacob et al. 2023). However, the fixed dose of the lactate infusion and the limited number of follow‐up assessments conducted in this particular study (Coco et al. 2009) does not preclude the fact that peripheral lactate concentration and cognitive performance change as a function of time, with larger benefits of higher blood lactate concentrations on cognitive performance at later follow‐ups (e.g., ≥ 10 min, as observed in other “real” physical exercise studies; Ballester‐Ferrer et al. 2022; Herold et al. 2022; Tsukamoto et al. 2016). The latter assumption is supported by evidence that a negative effect of lactate infusion on attention was only present at the 5‐min follow‐up when the peripheral blood lactate concentration had reached its peak. In contrast, such an effect was absent at the 10‐min follow‐up when the peripheral blood lactate declined to a level comparable to baseline (Coco et al. 2009).

Another study used a 120‐min lactate clamp intervention among older adults with or without cognitive impairments, starting with lactate infusion of ~2.6 mg/kgBW/min at rest, which was adjusted throughout the clamp to obtain a target blood‐lactate concentration of 4–5 millimolar (Kemna et al. 2025). Cognitive performance was assessed at baseline and at 90 min, while Alzheimer's Disease‐related blood markers were measured several times throughout the intervention period (i.e., baseline and at 10, 20, 30, 45, 60, 75, 90, and 120 min; Kemna et al. 2025). The authors observed pre‐ to posttest improvements in processing speed in both cohorts and in global cognition in cognitively healthy older adults (Kemna et al. 2025). Concerning the Alzheimer's disease‐related blood markers (i.e., pTau217, pTau181, brain‐derived tau, neurofilament light chain, and glial fibrillary acidic protein), a significant decrease from pretest to the follow‐up assessment at 120 min was observed in the whole cohort, except for total tau and brain‐derived neurotrophic factor (Kemna et al. 2025). In contrast to the findings of the latter study, a third study, utilizing a lactate clamp, provides evidence for a causal relationship between changes in peripheral blood lactate and brain‐derived neurotrophic factor (BDNF) concentrations in healthy young adults (Schiffer et al. 2011). In particular, to investigate the effect of blood lactate changes on plasma BDNF levels, eight healthy young adults underwent a lactate clamp intervention at rest wherein they received intravenous 4 molar sodium‐lactate solution at a progressively upregulated infusion rate. This procedure began with an infusion rate of 0.01 mL/kgBW/min in the first 3 min, and increased subsequently by 0.01 mL/kgBW/min every 3 min up to 0.08 mL/kgBW/min in the 24th minute (Schiffer et al. 2011). It was observed that the lactate clamp intervention at rest led to a significant postintervention increase in plasma BDNF levels, with a decline towards baseline levels at follow‐up assessments conducted 24 and 60 min after the infusion period (Schiffer et al. 2011). The findings of Schiffer et al. (2011) differ from those of Kemna et al. (2025), who did not observe changes in BDNF concentrations after a lactate clamp intervention. The contrasting findings are perhaps related to heterogeneity of sample characteristics (e.g., age and health status; healthy young adults versus older adults with or without cognitive impairments) or in study procedures (i.e., duration of lactate infusion; 24 versus 120 min). However, the observations of Schiffer and colleagues (Schiffer et al. 2011) mirror, at least partly, those of “real” acute physical exercise studies, showing an association between changes in peripheral blood lactate concentration and serum BDNF levels in response to 30 min of cardiorespiratory exercise in healthy young adults (Ferris et al. 2007). Here, results also demonstrated a return of acute physical exercise‐elevated BDNF levels to baseline 15–60 min after exercise cessation (for review see Dinoff et al. 2017). The finding that peripheral blood lactate concentration is likely to be causally related to BDNF expression (Schiffer et al. 2011), depending on modifiers (e.g., age, health status, and dose of lactate infusion), is practically relevant. This is especially true in healthy young adults, where the acute physical exercise‐related increase in serum BDNF has been associated with better immediate (i) short‐term learning (Winter et al. 2007) and (ii) cognitive flexibility performance (Hwang et al. 2016). In addition to its role in modulating BDNF, lactate also appears to influence other exercise‐sensitive growth factors. Evidence from human studies indicates that elevated peripheral blood lactate is positively correlated with changes in insulin‐like growth factor 1 (IGF‐1) and vascular growth factor (VEGF) concentrations (Kujach et al. 2020). Mechanistically, lactate can stimulate IGF‐1 expression through activation of the somatotropic axis and, as shown in animal studies, can promote VEGF synthesis and angiogenesis via hydroxycarboxylic acid receptor 1 (HCAR1) activation (Morland et al. 2017; Salgueiro et al. 2014). These observations suggest that blood lactate can act as a broader metabolic signal molecule influencing brain adaptations on multiple levels, reinforcing the utility of lactate infusion or clamp as promising paradigms (e.g., “physiologically informed” control conditions) to advance our understanding of why physical exercise can benefit cognition.

Taken together, the current body of evidence suggests that using lactate infusion or lactate clamp protocols as a “physiologically informed” control condition to mimic exercise‐related increases is a promising, yet underutilized, approach to complement results of correlational analyses (see Figure 1B). That said, it must be stressed that these methods require proper screening and monitoring (i.e., to avoid negative health events, such as panic attacks; Cowley and Arana 1990; Margraf et al. 1986), and are typically invasive (e.g., via intravenous infusion; Coco et al. 2009; Schiffer et al. 2011); however, noninvasive alternatives are available (e.g., via a drinking beverage entailing a lactic acid solution; Cho et al. 2020) and even in special populations (e.g., older adults with cognitive impairment) no intervention‐related adverse events were reported when using lactate infusion/clamp as an experimental manipulation (Kemna et al. 2025). These additional requirements can be resource‐intensive and can be considered a limitation. Despite this, these approaches have the potential to allow for more robust causal inferences as to whether and to what extent changes in peripheral blood lactate (e.g., in response to acute physical exercise) contribute to postexercise cognitive performance improvements, including the physiological mechanisms driving those alterations.

### 2.3 Passive Exercise

Passive exercise has been recently defined as a planned, structured, and repetitive form of passive movement designed to promote specific health outcomes (Tari et al. 2025). Passive movements are any externally driven limb or body movement that do not require voluntary muscle contraction (Trinity and Richardson 2019). In the context of brain health, including cognition, passive exercise is an understudied modality (Tari et al. 2024). However, as we will show below, some studies report promising findings.

Several studies, using a within‐subject design, investigated the effect of an acute bout of 20 min of passive exercise (i.e., passive cycling via a mechanically driven flywheel) on pre‐ and post‐condition inhibitory control in healthy young adults (Dalton et al. 2024; Jeyarajan et al. 2024; Shirzad et al. 2022; Tari et al. 2023). These studies compared passive exercise to time‐matched active exercise and resting control conditions, respectively. Compared to seated rest, passive exercise produced a positive effect on EF when performance was probed immediately after exercise cessation (Dalton et al. 2024; Jeyarajan et al. 2024; Shirzad et al. 2022; Tari et al. 2023). Notably, however, the passive exercise EF benefit did not persist up to 30 min postexercise, whereas active exercise showed a benefit at this timepoint (Tari et al. 2023). Moreover, passive exercise induced a significant increase in CBF compared to seated rest, albeit this was less pronounced than the increase observed during active physical exercise (Jeyarajan et al. 2024; Shirzad et al. 2022; Tari et al. 2023). In addition, passive but not active physical exercise improved EF when administered during exercise (Dalton et al. 2024). Another two studies, using a within‐subject cross‐over design with pretest‐posttest comparison, used passive exercise as a control condition for active physical exercise (i.e., cycling) while investigating changes to EF (i.e., inhibitory control and working memory) in healthy young adults (Weng et al. 2015) or functional brain activity patterns in healthy younger and older adults (Weng et al. 2017). Those studies observed that a 30‐min bout of moderate‐intensity active physical exercise, compared to passive exercise, led to (i) a postexercise improvement in working memory, but not inhibitory control, in young adults (Weng et al. 2015), and (ii) a postexercise increase in synchrony among brain regions associated with affect‐reward, hippocampal‐cortical, and executive‐control networks in healthy younger and older adults (Weng et al. 2017).

Taken together, these findings suggest that improvements in cognitive performance and functional brain activity patterns after a single bout of active exercise are related to some, yet‐to‐be‐fully‐defined, processes associated with voluntary muscle activation. However, it is likely that brain changes are not entirely dependent on these alterations because passive exercise, which does not require voluntary muscle activation because limb or body movements are driven by an external force (Tari et al. 2025), also improves cognitive performance (Dalton et al. 2024; Jeyarajan et al. 2024; Shirzad et al. 2022; Tari et al. 2023). Thus, using passive exercise as a “physiologically informed” control condition for active exercise (i.e., cycling) can be considered a promising approach to determine the amount of variance in postexercise cognitive performance changes attributable to active and metabolically demanding muscle contractions. These results can be further compared to interventions with a comparable range of limb movements but without associated respiratory or metabolic demands. Moreover, although passive exercise can provide a relatively rigorous “physiologically informed” control condition for active exercise, it must be acknowledged that passive exercise is largely limited to a singular exercise type (i.e., cycling) and does not include the broad range of movement parameters associated with active exercise modalities (e.g., running, walking, cross‐country skiing, etc.).

### 2.4 Motor Imagery

Another “physiologically informed” control condition to account, at least partly, for the demands posed by physiological processes involved in motor control is motor imagery. Motor imagery is the mental process of imagining a movement without physically performing it (Milton et al. 2008; Moran et al. 2012; Munzert and Zentgraf 2009) and provides the advantage over passive exercise in that it is not limited to a specific type of movement.

A systematic review with meta‐analysis showed that motor imagery recruited premotor, parietal, and somatosensory areas in a manner comparable but not identical to actual motor execution (for review see Hardwick et al. 2018). More specifically, motor imagery leads to higher activation in the dorsolateral prefrontal, premotor, and parietal cortices (e.g., bilateral presupplementary motor area, bilateral dorsal premotor cortex, left ventral premotor cortex, and left inferior and superior parietal cortex) compared to motor execution, whereas motor execution is associated with a higher activity in motor, sensorimotor (e.g., left primary motor and somatosensory cortex, and bilateral ventral premotor cortex) and subcortical (e.g., left putamen and cerebellum) regions compared to motor imagery (for review see Hardwick et al. 2018). More detailed information on theories concerning the mechanisms associated with motor imagery can be found elsewhere (Hurst and Boe 2022; O'Shea and Moran 2017).

To our knowledge, and at the time of writing, there exist only two within‐subject, cross‐over studies which used motor imagery as an intervention or control condition (e.g., to acute physical exercise) while assessing pretest–posttest cognitive outcomes (Johnson et al. 2019; Wieland et al. 2022). These studies compared single bouts of time‐matched “real‐executed” exercise versus first‐person imagined exercise in healthy young adults (Johnson et al. 2019; Wieland et al. 2022). One of these studies did not observe a significant difference between conditions on paired‐associative learning task performance (i.e., probed immediately and 10 min after intervention cessation) following 10‐min intermittent sprints (Johnson et al. 2019), whereas the other study reported improved attentional performance (i.e., probed 10 min after the intervention cessation) following a 25‐min moderate‐intensity cardiorespiratory “real executed” exercise condition compared to the imagined session (Wieland et al. 2022).

Although it is premature to make strong conclusions concerning the effects of acute motor imagery on cognitive performance due to the limited number of studies, the above studies highlight the potential of motor imagery as a “physiologically informed” control condition (i.e., to mirror to a certain extent the motor control‐related brain activity patterns), especially for more complex movements. Based on motor imagery's noninvasive nature and ease of application, there is potential for a more widespread application of this “physiologically informed” control condition in future acute physical exercise cognition studies. In doing so, potential caveats such as imaging perspective and type, task complexity of the to‐be‐imaged task, duration of the motor imagery session (Rozand et al. 2016), and participants' motor imagery ability (Madan and Singhal 2012; Olsson and Nyberg 2010) should be considered. This is because these moderators can be associated with differences in brain activation patterns (Furuta et al. 2024; Guillot et al. 2008; Lebon et al. 2012; Menicucci et al. 2020; Milton et al. 2008; Williams et al. 2012) for which more detail is provided elsewhere (Guillot and Collet 2008; Holmes and Collins 2001). Furthermore, when designing or interpreting findings of studies using motor imagery as a “physiologically informed” control condition, it should be considered that motor imagery can induce acute activation of the autonomic nervous system (e.g., an increase in heart or respiratory rate; Beyer et al. 1990; Decety et al. 1991, 1993; Oishi and Maeshima 2004), which is typically less pronounced than in “real executed” exercise (e.g., Johnson et al. 2019; Wang and Morgan 1992).

An alternative to motor imagery is action observation, which can include watching movements (e.g., exercise) without actually performing them (Hardwick et al. 2018; Olsson and Nyberg 2010). This modality shows some overlap with motor execution, but includes distinct brain activation patterns compared to actual exercise performance (Hardwick et al. 2018). Both motor imagery and action observation could be used as “physiologically informed” control conditions to account for the physiological processes of motor control (e.g., to isolate the effect of specific brain activation patterns required for movement execution).

### 2.5 Electrical Muscle Stimulation

Electrical muscle stimulation (EMS), also referred to as neuromuscular electrical stimulation, is a technique that uses transcutaneous electrical impulses delivered through electrodes attached to the skin over specific muscles or muscle groups to elicit involuntary muscle contractions, with the participants typically in a static, often supine, position (Blazevich et al. 2021; Enoka et al. 2020; Maffiuletti et al. 2011; Vanderthommen and Duchateau 2007). It is worth noting that EMS occurs without voluntary motor‐related efferent commands from the central nervous system. Despite the lack of central efferent nervous system involvement, this technique can produce (i) a central contribution due to the electrically evoked sensory volley (for reviews see Bergquist et al. 2011; Collins 2007), and (ii) increased activity in some brain regions, including sensorimotor regions (Maffiuletti 2010; Smith et al. 2003). Although EMS is an established and widely used technique in the broader field of exercise and rehabilitation science (Enoka et al. 2020; Gondin et al. 2011; Kashyap et al. 2023; Langeard et al. 2017; Moritani 2021; Nishida et al. 2016; Nussbaum et al. 2017; Sanchez et al. 2023; Vanderthommen and Duchateau 2007), its application to modulate cognition is still in its infancy, especially when narrowing the scope to studies using this experimental manipulation as a control condition for acute physical exercise. Moreover, EMS can be considered an interesting technique to mimic, at least to a certain extent, broader physiological responses related to muscle activation in a resting condition, although those electrically induced muscle contractions are physiologically different from voluntary ones (for reviews see Bergquist et al. 2011; Bickel et al. 2011; Gregory and Bickel 2005; Maffiuletti 2010). As such, the technique may provide a relevant protocol to provide a better mechanistic understanding of an exercise‐cognition interaction, with the potential mechanisms of EMS on the brain having been reviewed elsewhere in more detail (for review see Descollonges, Chaney, et al. 2024). In support of this assumption, we outline two within‐subject studies with a randomized cross‐over design (Ando et al. 2021; Descollonges, Marmier, et al. 2024). Here, EMS was applied to large muscle groups (e.g., abdomen, and bilaterally to gluteal, thigh, and leg muscles) of healthy young adults for 20 (Ando et al. 2021) to 25 min (Descollonges, Marmier, et al. 2024) at individual maximum tolerable intensity. Verbal learning and selective attention (i.e., Stroop congruent condition) performance was assessed immediately postexercise, and results were compared to a seated rest condition (Descollonges, Marmier, et al. 2024). Overall, the studies found in the samples of healthy young adults (i) an intervention‐induced increase in CBF (Ando et al. 2021; Descollonges, Marmier, et al. 2024), an increased cortical hemodynamic response in the left prefrontal cortex (Descollonges, Marmier, et al. 2024), and postintervention cognitive improvements (Descollonges, Marmier, et al. 2024). Despite these promising findings, studies comparing acute EMS to acute exercise are relatively scarce. One within‐subject randomized cross‐over study with pretest‐posttest comparisons investigated the effects of 30 min of EMS on EF and peripheral blood biomarkers of healthy young adults (Miyamoto et al. 2018). Muscle stimulation was delivered to both legs, and the intervention was time‐matched to a session of moderate‐intensity continuous cardiorespiratory exercise and a seated resting control (Miyamoto et al. 2018). This study showed that EMS triggered an increase in peripheral plasma BDNF levels equivalent to that observed in response to moderate‐intensity cardiorespiratory exercise; however, no statistically significant effect on EF was observed (Miyamoto et al. 2018).

Overall, the limited number of available studies does not allow for robust conclusions; however, that EMS can induce the release of peripheral plasma BDNF comparable to acute exercise suggests that this type of experimental manipulation represents a promising ‘“physiologically informed”, yet underutilized, alternative to traditional control conditions. Applying EMS may enable the better isolation of broader physiological responses triggered by voluntary muscle activation and their influence on postexercise cognitive changes. However, the physiological differences between voluntary muscle activation patterns and those evoked by EMS (e.g., muscle activation is spatially fixed, superficial, synchronous, incomplete, and more fatiguing when using EMS; for reviews see Bergquist et al. 2011; Bickel et al. 2011; Gregory and Bickel 2005; Maffiuletti 2010) should be considered when planning a study protocol. Another disadvantage is that EMS may induce discomfort, depending on the participant's pain sensitivity and the intensity of the electrical stimulation. Future studies using this experimental manipulation as a “physiologically informed” control condition should aim to mimic muscle activation patterns recorded via electromyography during single bouts of physical exercise. This should be done while also adhering to safety and other methodological recommendations concerning the application of EMS to avoid adverse health‐related events (for reviews see Gobbo et al. 2011; Maffiuletti 2010; Maffiuletti et al. 2018; Nishida et al. 2016; Nussbaum et al. 2017; Stöllberger and Finsterer 2019).

## 3 Discussion

An appropriate control condition/group is crucial for making robust causal inference concerning the effect of an intervention and the underlying mechanisms (Green et al. 2014, 2019; Herold et al. 2021; Pontifex et al. 2019). Currently, the selection of control conditions/groups in the exercise‐cognition field is primarily centered around (i) study design, (ii) level of energy expenditure, and (iii) level of cognitive engagement. As such, the broader exercise‐cognition field has not broadly considered a “physiological” perspective for determining an appropriate control group/condition. By discussing selected examples, particularly hypercapnia, lactate infusion/clamp, passive exercise, motor imagery, and EMS, we show how “physiologically informed” control conditions may serve as a valuable tool for advancing our understanding of the physiological mechanisms that drive the effects of physical exercise on cognitive performance (Erickson et al. 2019; Stillman et al. 2016, 2020).

Given that the effects of physical exercise on cognition are likely to be mediated by changes on multiple physiological levels (Herold, Törpel, et al. 2019; Stillman et al. 2016, 2020), we recommend collecting multiple psychophysiological measures. These will depend on the aims and context of research and allow the field to gain a more comprehensive understanding of how specific physiological candidate mechanisms and the way in which they interact contribute to acute physical exercise‐related cognitive improvements. Concerning single bouts of physical exercise, we suggest centering such a multimodal assessment around a method that (i) quantifies functional brain changes, such as CBF estimated via transcranial Doppler ultrasound (e.g., as used in several studies; Dalton et al. 2024; Jeyarajan et al. 2024; Shirzad et al. 2022; Tari et al. 2023) or cerebral hemodynamics via functional near‐infrared spectroscopy (for reviews see Herold, Gronwald, et al. 2020; Herold et al. 2018), and (ii) simultaneously record, if possible, biochemical changes (e.g., using pupillometry to quantify changes in pupil size as a proxy for changes in the noradrenergic system; for reviews see Kuwamizu, Yamazaki, Suwabe, et al. 2025; Zou et al. 2023). Typically, physiological responses during acute physical exercise are manifold and interactive. Thus, mimicking those physiological responses and their interaction to the fullest extent is challenging, even with sophisticated control conditions. To assess the putative impact of specific physiological pathways on cognition mandates that future acute exercise‐cognition studies include a wide range of potentially relevant physiological measures. Recording a wide range of potentially relevant physiological measures is not only useful for elucidating the candidate physiological pathways that drive acute physical exercise‐related cognitive changes but also necessary to control how well “physiologically informed” control conditions have actually mimicked the acute physical exercise‐related physiological response(s). Controlling such intervention‐related physiological changes is highly relevant to interpreting findings because specific types of “physiologically informed” control conditions may influence cognition through pathways other than those primarily stimulated by acute physical exercise (e.g., pain‐related arousal changes when lactate infusion/clamp or EMS is used).

Overall, “physiologically informed” control conditions, together with “traditional” controls, constitute a form of methodological triangulation in which multiple modes of experimental manipulation (see Figure 1) are used to provide a more comprehensive understanding of a particular phenomenon (Berg et al. 2025). Although such a triangulation approach is not without limitations (e.g., more complex and resource‐consuming; Herold, Gronwald, et al. 2026), it may allow for accounting for the issues summarized as the “fat‐handed”^2^ phenomenon (Bailey et al. 2024; Eronen 2020; Scheines 2005) by enabling a more rigorous isolation of physiological candidate mechanisms that might explain the effects of physical exercise on cognition. Such a relatively rigorous isolation of physiological candidate mechanisms is often not feasible by manipulating exercise variables to adjust the dose of (acute) physical exercise. For example, peripheral blood lactate concentrations can also be manipulated by adjusting exercise intensity, with higher intensities typically leading to increased peripheral blood lactate concentrations (e.g., for reviews Faude et al. 2009; Goodwin et al. 2007). However, increasing exercise intensity also simultaneously increases CBF (e.g., from light to moderate intensity; for reviews see Ogoh and Ainslie 2009; Smith and Ainslie 2017) and constitutes another physiological candidate mechanism for postexercise cognitive improvements (Pontifex et al. 2019). From the perspective of making robust causal inference regarding postexercise cognitive improvements, a simultaneous change in several physiological candidate mechanisms is problematic (i.e., “fat‐handed” phenomenon; Bailey et al. 2024; Eronen 2020; Scheines 2005). Stated differently, while comparing exercise conditions with different “doses” (e.g., designed to induce specific changes in peripheral blood lactate concentration) can be a valuable approach to addressing particular research questions, it does not allow for the rigorous isolation of specific physiological mechanisms in a way that might be possible through “physiologically informed” control conditions. This can hinder how mechanistic research questions can be appropriately addressed. Therefore, considering “physiologically informed” control conditions (i.e., mimicking specific acute physical exercise‐induced responses without actually engaging in acute physical exercise) as a complement to traditional approaches can enable stronger causal inference about underlying physiological mechanisms and likely advance knowledge of the mediators of the exercise‐cognition link.

In this context, it is worth noting that such experimental manipulations (e.g., lactate infusion or clamp) can also be used *during* acute physical exercise, with a promising potential to provide complementary evidence compared to their application at rest. For example, lactate infusion or clamps could be used to mimic blood lactate concentrations of high‐intensity exercise while actually performing light‐intensity exercise. Contrasting a high‐intensity exercise condition/group to a light‐intensity exercise condition/group matched for other exercise characteristics (e.g., duration) and blood lactate response (i.e., through a lactate infusion or clamp), can be a valuable approach to assess whether postexercise cognitive performance changes are driven by alterations in blood lactate concentration (i.e., in case of comparable condition−/group‐related changes in cognitive performance), or other physiological mechanisms (e.g., CBF) sensitive to exercise intensity (i.e., in case of higher cognitive benefits in the high‐intensity exercise condition/group). As discussed elsewhere in more detail (Herold, Müller, et al. 2019), another valuable approach to advance our understanding of whether blood lactate acts as a mediator of postexercise cognitive performance changes is the “clamping” of the blood lactate concentration during exercise (i.e., as a marker of *internal load*, which is the psychophysiological response to external load; Gronwald et al. 2020; Herold, Müller, et al. 2019; Impellizzeri et al. 2019) through a purposeful adjustment of the *external load* (i.e., work completed by the participant independent of individual characteristics and influencing environmental conditions; Gronwald et al. 2020; Herold, Müller, et al. 2019; Impellizzeri et al. 2019). Inducing comparable blood lactate concentrations across participants via a tailored exercise prescription is especially useful for elucidating the role of potential effect modifiers (e.g., individual characteristics, such as baseline cognitive performance level), as high interindividual heterogeneity in cognitive outcomes, while showing comparable blood lactate concentrations, may point towards pronounced effects of such factors. Besides lactate infusion, such experimental manipulations during physical exercise can also include other approaches (e.g., EMS during light‐intensity exercise to mimic muscle activation patterns of high‐intensity exercise), with several studies in other application scenarios (e.g., in stroke rehabilitation (Descollonges et al. 2025) or to investigate acute effects on cognition (Ando, Fujimoto, et al. 2024; Ando, Ishioka, et al. 2024; Descollonges et al. 2026)), have documented that using EMS during acute physical exercise is feasible and relatively well‐tolerated. Furthermore, this may also include pharmacological intervention approaches to manipulate neuromodulatory systems, such as norepinephrine or dopaminergic systems (e.g., as conceptualized in the catecholamine hypothesis (Cooper 1973) and relevant extensions (McMorris 2021)), via specific drugs (e.g., a dopamine reuptake inhibitor such as methylphenidate, a norepinephrine reuptake inhibitor such as reboxetine, or a norepinephrine‐dopamine reuptake inhibitor such as bupropion), if ethically acceptable. Such pharmacological intervention approaches, which increase synaptic transmission of those neurochemicals by blocking reuptake transporters, have been successfully used in human exercise research, especially those concerned with elucidating neurophysiological mechanisms of fatigue (e.g., Arauz et al. 2026; Connell et al. 2017a, 2017b; Klass et al. 2012, 2016, 2018; Roelands, Goekint, et al. 2008; Roelands, Hasegawa, et al. 2008; Roelands et al. 2012). Those pharmacological interventions (e.g., norepinephrine and/or dopamine reuptake inhibitors) showed an influence on tasks, particularly pro‐ and antisaccade task (Connell et al. 2017a, 2017b) used for probing EF (Hutton 2004; Munoz and Everling 2004), that are widely used to assess the effects of acute physical exercise on cognition (for review see Zou et al. 2023). The cognitive effects of such pharmacological interventions (e.g., using a dopamine reuptake inhibitor such as methylphenidate), which are modulators of cognitive performance in healthy populations (for review see Linssen et al. 2014) and those with a disorder (e.g., attention‐deficit/hyperactivity disorder; for reviews see Mckenzie et al. 2022; Pievsky and McGrath 2018; Tamminga et al. 2016), might be explained by their impact on neuromodulatory systems (e.g., dopaminergic system) that influence activation patterns in brain areas related to motor control (e.g., motor cortex during (King et al. 2017) and after (King et al. 2018) acute physical exercise exposure), or those relevant for higher‐order cognitive processes (e.g., prefrontal cortex (Farias et al. 2019; Lourenço et al. 2026; Mueller et al. 2014) as neural correlate of EF (Funahashi and Andreau 2013)). Given that changes in dopaminergic and noradrenergic systems are potential candidate mechanisms that drive acute physical exercise‐related cognitive benefits (for reviews see Hou et al. 2024; Pontifex et al. 2019), studying their influence through pharmacological interventions, as a type of “physiologically informed” control condition, can be considered a promising approach to advance our mechanistic understanding of the acute exercise‐cognition link. We recommend those courses of action, if those interventions are ethically acceptable. This idea is reinforced by the evidence suggesting that acute physical exercise‐related EF benefits are associated with brain activation changes in the prefrontal cortex (for review see Herold et al. 2018); a brain area especially sensitive to both dopaminergic and noradrenergic modulations (Arnsten 1998; Arnsten and Li 2004; Xing et al. 2016). In a broader sense, a “physiologically informed” approach may also prove useful to understand the longevity of the aftereffects of intense physical exercise. As a complement to the traditional approach that uses fixed time intervals for postexercise cognitive testing (e.g., 5, 10, 15, or 20 min after exercise cessation), the “physiologically informed” approach schedules the timing based on the postexercise patterns of relevant physiological responses (e.g., trajectories of CBF (Kennedy et al. 2022; Mulser and Moreau 2023)), cerebral hemodynamics (Ohyanagi et al. 2018; Oyanagi and Tsubaki 2021; Oyanagi et al. 2016; Qin et al. 2024; Tsubaki et al. 2017, 2018, 2020, 2021; Tsubaki, Takai, Kojima, et al. 2016; Tsubaki, Takai, Oyanagi, et al. 2016), or pupil size (Kuwamizu, Yamazaki, Aoike, et al. 2025), with the latter as an indicator of the locus coeruleus‐norepinephrine system (Kuwamizu, Yamazaki, Suwabe, et al. 2025; Zou et al. 2023). In such a scenario, cognitive assessments would be conducted when the postexercise upregulation of specific neuromodulatory systems is optimal (i.e., highest benefit) or suboptimal (i.e., lower benefit; serving as “proof‐of‐concept” control condition), with an optimal state for most physiological parameters has yet to be identified. Since our knowledge of the neurophysiological mechanisms that influence how long the cognitive aftereffects of acute physical exercise last is quite limited (for reviews see Herold, Zou, et al. 2025; Pontifex et al. 2019), applying this approach in future studies may offer valuable insights and guide the development of potentially more effective exercise prescription approaches (e.g., personalized exercise prescription). Complementary to other personalized exercise prescription approaches (Barha et al. 2017, 2021a, 2021b; Gronwald et al. 2020; Herold, Müller, et al. 2019; Herold, Törpel, et al. 2020; Moreau and Wiebels 2024; Pickering and Kiely 2018; Solis‐Urra et al. 2024), our approach proposed above might contribute to the development of more personalized ones by aiding (i) the refinement of existing theories (e.g., catecholamine hypothesis (Cooper 1973) and relevant extensions (McMorris 2021)), (ii) to the explanation of why behavioral benefits from exercise often are substantially smaller after a certain period (e.g., > 15 min after exercise cessation; Chang et al. 2012), and (iii) the identification of the ideal timing (i.e., exercise density; Herold, Owen, et al. 2025; Herold, Zou, et al. 2025, 2026; Manci et al. 2024) for repeating acute physical exercise bouts to prolong their positive cognitive effects.

Last, we wish to stress that the proposed approach should not be confused with “*exercise mimetics*” as an intervention aiming to harness the benefits of physical exercise as a therapeutic (e.g., in the form of an “exercise (poly‐)pill”; Fan and Evans 2017; Gubert and Hannan 2021; Hannan 2025; Hawley and Holloszy 2009). It is our opinion that this approach is far from being ready to replace all the health benefits of physical exercise (Booth and Hawley 2015; Booth and Laye 2009; Hawley et al. 2021), and such a reductionistic approach underappreciates the complexity of the extensive range of physiological responses triggered by physical exercise.

## 4 Conclusions

An appropriate control condition/group is not only a key factor to make more robust causal inferences regarding the effects of specific physical exercise interventions on human behavior, but also for the mechanisms driving the putative intervention‐related effects. The physiological mechanisms driving the positive effects of physical exercise on cognition remain to be fully understood. To advance our understanding of those mechanisms, we propose that future studies should consider “physiologically informed” control conditions in which experimental manipulations are used to mimic the effects of physical exercise without engaging in physical exercise. Such “physiologically informed” control conditions can complement “traditional” controls and strengthen the causal inferences they provide. These conditions can provide a more appropriate “contrast condition” which allows for a more rigorous isolation of physiological mechanisms related to the physical exercise‐induced changes in cognitive performance. Given that this approach holds serious implications for the ability of the exercise‐cognition research field to move forward with the mechanistic understandings of physical exercise‐induced cognitive performance changes, we advocate for its more widespread application in future studies in this field.

## Author Contributions

**Fabian Herold:** conceptualization, writing – original draft, visualization. **Benjamin Tari:** writing – review and editing. **Sylwester Kujach:** writing – review and editing, visualization. **Olivier Dupuy:** writing – review and editing. **C. Shawn Green:** writing – review and editing. **Thomas Gronwald:** writing – review and editing. **Matthew Heath:** writing – review and editing. Fabian Herold is responsible for the overall content as guarantor. All authors read and approved the final manuscript.

## Funding

The authors have nothing to report.

## Ethics Statement

The authors have nothing to report.

## Consent

The authors have nothing to report.

## Conflicts of Interest

The authors declare no conflicts of interest.

## Data Availability

Data sharing not applicable to this article as no datasets were generated or analysed during the current study.

## Endnotes

^1^
For a more detailed discussion of the advantages and disadvantages of other study designs in acute exercise‐cognition studies, which is beyond the scope of this article, the reader is referred to the following literature: Herold et al. (2021); Pontifex et al. (2019).

^2^
The term “fat‐handed” was coined in analogy to accidentally knocking over chess pieces while reaching to make the next move because of a “fat hand” (Scheines 2005). In less metaphorical terms, this means that an (exercise) intervention does not only cause a change in a single variable, as typically intended, but also simultaneously triggers non‐intended changes in other variables that influence a specific outcome (for reviews see Bailey et al. (2024); Eronen (2020)).
